# An M29 Aminopeptidase from *Listeria Monocytogenes* Contributes to In Vitro Bacterial Growth but not to Intracellular Infection

**DOI:** 10.3390/microorganisms8010110

**Published:** 2020-01-13

**Authors:** Xian Zhang, Chiyu Guan, Yi Hang, Fengdan Liu, Jing Sun, Huifei Yu, Li Gan, Huan Zeng, Yiran Zhu, Zhongwei Chen, Houhui Song, Changyong Cheng

**Affiliations:** 1 Key Laboratory of Applied Technology on Green-Eco-Healthy Animal Husbandry of Zhejiang Province, China-Australian Joint Laboratory for Animal Health Big Data Analytics, Zhejiang Provincial Engineering Laboratory for Animal Health Inspection & Internet Technology, College of Animal Science and Technology & College of Veterinary Medicine of Zhejiang A&F University, Zhejiang A&F University, Lin’an 311300, China; zhangxian073@163.com (X.Z.); jingsun@zafu.edu.cn (J.S.);; 2 Jixian Honors College of Zhejiang A&F University, Zhejiang A&F University, Lin’an 311300, China; zyr20090708@163.com

**Keywords:** aminopeptidase, the M29 family, *Listeria monocytogenes*, bacterial growth, infection

## Abstract

Aminopeptidases that catalyze the removal of N-terminal residues from polypeptides or proteins are crucial for physiological processes. Here, we explore the biological functions of an M29 family aminopeptidase II from *Listeria monocytogenes* (LmAmpII). We show that LmAmpII contains a conserved catalytic motif (EEHYHD) that is essential for its enzymatic activity and LmAmpII has a substrate preference for arginine and leucine. Studies on biological roles indicate that LmAmpII is required for in vitro growth in a chemically defined medium for optimal growth of *L. monocytogenes* but is not required for bacterial intracellular infection in epithelial cells and macrophages, as well as cell-to-cell spreading in fibroblasts. Moreover, LmAmpII is found as dispensable for bacterial pathogenicity in mice. Taken together, we conclude that LmAmpII, an M29 family aminopeptidase, can efficiently hydrolyze a wide range of substrates and is required for in vitro bacterial growth, which lays a foundation for in-depth investigations of aminopeptidases as potential targets to defend *Listeria* infection.

## 1. Introduction

*Listeria monocytogenes* is a facultative intracellular bacterial pathogen that is capable of passing through the intestinal epithelial barrier, blood-brain barrier, or fetoplacental barrier into target organs and colonizing them, thereby infecting host cells [1,2]. In the process of continuously establishing infections by *L. monocytogenes*, in addition to the known classical virulence factors, such as listeriolysin O, phospholipases, and actin polymerization protein [2], the metalloprotease (Mpl) families also play a critical role. *L. monocytogenes* Mpl is a thermolysin-like protease that mediates the maturation of a broad-range phospholipase C, which contributes to the ability of this pathogen to escape from the vacuolar and grow intracellularly. Mpl is made as a proprotein that undergoes maturation by proteolytic cleavage of a N-terminal premature domain [3,4].

Aminopeptidases (APs), one of the Mpl groups, can catalyze the cleavage of N-terminal amino acids in proteins or peptides, releasing amino acid residues, preferably the hydrophobic residues [5]. These peptidases are widely distributed in bacteria, fungi, and other species with important physiological roles, such as protein maturation, protein turnover, hydrolysis of regulatory peptides, nitrogen nutrition, modulation of gene expression, support of the amino acids pool, and virulence factors, and thus are considered essential enzymes [5,6,7,8,9,10]. Based on the hierarchical and structure-based classification of the peptidases, APs are divided into clans, MA, MF, MG, MH, MN, and MQ in the MEROPS database (also termed proteases, proteinases, and proteolytic enzymes) [11,12], and most aminopeptidases are metalloenzymes, except for prolyl aminopeptidase (serine peptidases) and DmpA aminopeptidase (nucleophile hydrolase) [12]. The M29 family, one of the characterized aminopeptidase families, encompasses aminopeptidase S (AmpS) [13], aminopeptidase II (AmpII) [14], aminopeptidase T [15], PepS aminopeptidase [16], and their homologues in each member group. In addition to most of the studies focusing on the biochemical properties of the M29 family aminopeptidases, more researchers have increasingly been concentrating on the biological roles of this peptidase family. The aminopeptidase MesoAmp of the M29 family from *Mesorhizobium* has been found to play an important role in biofilm formation and osmotic stress tolerance [6]. The PepS from *Streptococcus thermophilus* has been demonstrated to play a pleiotropic role through its involvement in growth via nitrogen nutrition, as well as via other cellular functions or metabolisms [5]. In *L. monocytogenes*, an X-prolyl aminopeptidase (PepP) has been suggested to play a role in posttranslational activation of the major virulence regulator, PrfA via mediating recruitment of PrfA to the cytoplasmic membrane, a step identified as critical for PrfA activation [17]. Our previous study has demonstrated that *lmo1603* encodes a functional aminopeptidase T of the M29 family and plays an important role in the successful establishment of *L. monocytogenes* infection [8]. Additionally, we identified a putative AmpII (LmAmpII) that belongs to the M29 family, but the biological roles of which have not been characterized to date [18]. According to the MEROPS database [12], LmAmpII belongs to the metal-dependent M29 family that contains the highly conserved catalytic residues (EEHYHD). In this study, we aimed to further elucidate the functional and biochemical characteristics of LmAmpII and the data for the first time to demonstrate the interesting features and biological roles of AmpII in *Listeria* metabolism and infection. The study supports the potential of this enzyme for applications in biotechnological processes and lays a foundation for in-depth investigations of aminopeptidases as potential targets to defend *Listeria* infection.

## 2. Materials and Methods

### 2.1. Bacterial Strains, Plasmids, and Growth Conditions

We used the reference strain *L. monocytogenes* EGD-e. Escherichia coli (*E. coli*) DH5α was employed as the host strain for plasmids pET30a (+) (Novagen), integration vector pIMK2, and temperature-sensitive plasmid pKSV7 [19,20]. *E. coli* BL21 was used for prokaryotic protein expression. *L. monocytogenes* EGD-e and *E. coli* DH5α were cultured in brain heart infusion (BHI, Oxoid, Basingstoke, England) and Luria–Bertani broth (LB, Oxoid) medium, respectively, at 37 °C with shaking. When indicated, antibiotics were used at the following concentrations: chloramphenicol (10 μg/mL) and kanamycin (50 μg/mL).

### 2.2. Cell Fractionation and Protein Localization Analysis

*L. monocytogenes* stocks were prepared in BHI media containing 50% (*v*/*v*) glycerol and kept at −80 °C. Bacteria were recovered on BHI agar by using a sterile loop and grown overnight at 37 °C, and a single colony was picked and grown in 5 mL BHI broth overnight at 37 °C. *L. monocytogenes* EGD-e were diluted (1:50) into 500 mL fresh BHI broth, and bacteria were grown to an optical density (OD. 600 nm) of 1.0. For secreted protein (SP) isolation: The bacterial pellet and supernatant were harvested by centrifugation at 13,000× *g* for 20 min at 4 °C. The supernatant was filtered through a 0.22 μm filter (Merck, Darmstadt, Germany) to remove any remaining bacterial cells. Trichloroacetic acid (TCA) was added to the supernatant to reach a final concentration of 10% (*v*/*v*) and sample incubated overnight on ice and then centrifuged at 12,000× *g* for 5 min. Finally, the precipitates washed with ice-cold acetone were resuspended in a SDS-PAGE sample buffer (5% SDS, 10% glycerol, and 50 mM Tris-HCl, pH 6.8). Samples were boiled for 5–10 min and stored at −20 °C for further use. For whole cell lysates (WCLs): The bacterial pellet was resuspended in 1 mL extraction solution (2% Triton X-100, 1% SDS, 100 mM NaCl, 10 mM Tris-HCl, 1 mM EDTA, pH 8.0) and lysed by using the homogenizer (Bertin, Provence, France). The pellet was then discarded and the supernatant was retained as the whole cell extract. For membrane proteins (MPs): The whole cell extract was ultra-centrifuged at 100,000× *g* for 1 h at 4 °C to obtain the membrane pellet that was then resuspended in a 1 mL extraction solution and finally ultra-centrifuged at 100,000× *g* for an additional 1 h. The resulting supernatant fractions were removed and the pellet that represents the membrane-containing fraction was kept at −20 °C before use. For cell wall proteins (WPs): Bacterial pellets were resuspended in 0.5% of the original culture volume of 10 mM PBS containing 2% (*w*/*v*) SDS for 30 min. Bacterial suspensions were centrifuged at 12,000× *g* for 10 min, and the supernatant containing bacterial cell wall proteins was filtered (0.22 μm) and the filtrate was subjected to the following experiments.

### 2.3. Generation of In-Frame Gene Deletion Mutant and Complemented Strain

*L. monocytogenes* gene deletion and complementation strategy was employed as described previously [21,22]. Upstream and downstream regions of the interest gene were overlapped using a homologous recombination strategy with an overlap extension PCR procedure (see the primers in Table 1) and cloned into the plasmid pKSV7. The recombinant plasmid was sequenced and electroporated into EGD-e competent cells. Transformants were grown at a non-permissive temperature (42 °C) in BHI broth (10 μg/mL chloramphenicol) to promote chromosomal integration [19]. The chromosomal integration was confirmed using PCR with the primer pair (LmAmpII-a-front/LmAmpII-d, Table 1). Thereafter, recombinant bacteria were cultured 10 to 30 times on BHI agar without any antibiotics at a permissive temperature (30 °C) to enable plasmid excision and curing. Finally, the recombinants that lost pKSV7 were identified as chloramphenicol-sensitive colonies and an allelic exchange was confirmed by PCR (LmAmpII-a-front/LmAmpII-d, Table 1) and Sanger DNA sequencing. The deletion mutant was designated as **Δ***LmAmpII*. For gene complementation, the complete ORF of *LmAmpII*, along with its native promoter region, was amplified using the primer pairs (Table 1) and cloned into the integrative plasmid pIMK2. The resulting plasmid was then electroporated into the competent mutant strain, according to the method previously described [23]. Specifically, overnight-grown *Listeria* were diluted (1:50) into 100 mL fresh BHI broth containing 0.5 M sucrose and cultured until reaching an OD at 600 nm of 0.2. The penicillin G was added with a final concentration of 10 μg/mL and the incubation continued for a further 2 h. Cells were harvested and washed using the electroporation buffer (1 mM HEPES in 0.5 M sucrose, pH 7.0). The final cell pellet was resuspended in 200 μL electroporation buffer and the bacterial competent was electroporated in 50 μL aliquot with 1 μg recombinant plasmid. The regenerated colonies were selected by plating on BHI agar containing kanamycin (50 μg/mL), and the complemented strain was designated as C**Δ***LmAmpII*.

### 2.4. Heterologous Overexpression and Protein Purification

The complete ORF of the *LmAmpII* gene was amplified and cloned into a pET-30a expression vector, submitted to DNA sequencing, and inserted into the expression host *E. coli* BL21. Bacteria harboring the recombinant plasmid were grown at 37 °C. When the bacterial absorbance reached an OD at 600 nm of 0.6, expression of the recombinant protein was induced by addition of isopropyl β-D-1-thiogalactopyranoside (IPTG, Sangon, Shanghai, China) to a final concentration of 0.4 mM, and the incubation continued at 30 °C for an additional 4 h. Bacterial cells were collected and lysed, and the supernatant was applied onto a nickel-chelated resin (Weishi-Bohui Chromtotec Co. Beijing, China). Proteins were eluted with a concentration gradient of imidazole (30–300 mM), and a protein concentration of the eluted fractions was determined by the BCA protein assay method. The purity of the recombinant protein was analyzed by Coomassie-stained 12% SDS-PAGE. 

### 2.5. Site-Directed Mutagenesis

To experimentally verify the predicted active sites of LmAmpII, a single site-directed mutant (E250A, E316A, H345A, Y352A, H378A, or D380A) was generated on the original expression vector using the primer pairs (Table 1). Template DNA was then removed by digestion with DpnI (TOYOBO (Shanghai) Co., Ltd., Shanghai, China) for 2 h at 37 °C. All mutants were sequenced to ensure that only the desired single mutations had been correctly introduced into the wild-type protein expression construct. The procedures for expression and purification of these variants were performed as described above for the wild-type protein.

### 2.6. Generation of Polyclonal Antibodies

Polyclonal antibodies against LmAmpII were generated in New Zealand white rabbits, according to a standard protocol [24,25]. Rabbits were initially immunized by subcutaneous injection of 500 μg protein antigens with equal volume of Freund’s complete adjuvant (Sigma-Aldrich). After two weeks, the rabbit was boosted subcutaneously three times with 250 μg protein antigen with an equal volume of incomplete Freund’s adjuvant (Sigma-Aldrich) at 10-day intervals. The rabbit was bled and serum was collected 10 days after the last injection.

### 2.7. Enzymatic Activity Assays

The aminopeptidase activity was measured by spectrophotometric detection via the hydrolysis of amino acids-*p*-nitroaniline (AAs-*p*NA) by continuously monitoring the release of *p*NA at 405 nm using the spectrophotometer reader Synergy H1 (BioTek) [26]. Unless otherwise indicated, the reaction was performed using 0.05 μM of purified LmAmpII or its variants in 25 mM Tris-HCl buffer (pH 7.4), containing 1 mM substrate at 37 °C in a final volume of 200 μL. Metal ion dependence of aminopeptidase was investigated by assaying the activity after pre-incubation of the purified enzyme (0.05 μM) in 25 mM Tris-HCl (pH 7.4) with 0.5 mM specified metal ions (Co^2+^, Mg^2+^, Fe^3+^, Zn^2+^, Mn^2+^, or Cd^2+^) at 37 °C, containing 1 mM Leu-*p*NA as the substrate. The relative enzyme activity was calculated from the control sample (without addition of any metal ion) set to 100%. To determine the reaction optimum pH for aminopeptidase activity, the purified enzyme (0.05 μM in 25 mM Tris-HCl, 37 °C) was incubated at different pHs (ranging from 3.5 to 11.5) for 1 h before addition of substrate (1 mM Leu-*p*NA). To assess the effect of temperature on the aminopeptidase activity, the enzymatic reactions took place at various temperatures ranging from 4 to 50 °C. The relative enzyme activities were calculated from the corresponding control samples set to 100%.

### 2.8. The Michaelis–Menten Kinetic Parameters

The Michaelis–Menten kinetic parameters were determined by incubating 0.05 μM purified enzyme with each AA-*p*NA substrate at different concentrations (ranging from 0 to 1000 μM). The reaction was initiated by adding the enzyme in 25 mM Tris-HCl (pH 7.4) containing the substrate at 37 °C. Enzymatic reaction rates (*v*_0_) versus substrate concentrations were fitted to a Michaelis–Menten equation I (*v*_0_ = *V*_max_ [S]/(*K*_m_ + [S]), as previously described [21]. The values *K*_m_, *V*_max_, and *K*_cat_ were calculated using the software GraphPad Prism 8.0 (GraphPad Software, La Jolla, CA, USA), where [S] is the substrate concentration, *v*_0_ the initial velocity, *V*_max_ the maximum velocity, and *K*_m_ the Michaelis–Menten constant. The *K*_cat_ value was then determined from *V*_max_ and the enzyme concentration [E] using the equation II (*K*_cat_ = *V*_max_/[E]) [21].

### 2.9. In Vitro Growth of L. monocytogenes in BHI Broth and Defined Medium

*L. monocytogenes* cells were grown overnight at 37 °C in BHI broth with shaking. Cultures were collected by centrifugation at 5000× *g* at 4 °C, washed twice in 10 mM PBS (pH 7.4) and the initial OD_600 nm_ was adjusted to 1.0 (about 10^9^ CFU/mL). Bacteria were diluted (1:100) in fresh BHI broth, or in a chemically defined medium for the optimal culture of *Listeria* [27], and incubated at 37 °C for an additional 12–26 h. The kinetic growth was measured on a 96-well plate and bacterial ODs were measured at a 1- or 2-h interval. 

### 2.10. Intracellular Growth in J774A.1 and RAW264.7 Macrophages

Intracellular growth was performed on the murine macrophages, J774A.1 and RAW264.7. Monolayers of macrophages were grown to 95–100% confluence in Dulbecco’s modified eagle medium (DMEM, Thermo Fisher), containing 10% fetal bovine serum (FBS, Hyclone). Stationary bacteria were washed twice and re-suspended in 10 mM PBS (pH 7.4) and then employed to infect cells for 30 min with a multiplicity of infection (MOI) at 0.05. The gentamicin at a final concentration of 50 μg/mL was added to kill extracellular bacteria for an additional 30 min. The infected cells incubated in DMEM, containing 5 μg/mL gentamicin and 10% FBS, were then lysed at 2, 6, or 12 h of incubation. Intracellular bacteria in lysates were enumerated by plating serial dilutions of homogenates on BHI agar plates.

### 2.11. Adhesion and Invasion in Caco-2 Cells

Adhesion and invasion of *L. monocytogenes* were performed as previously described [2,28]. Bacterial cultures grown overnight were washed twice and resuspended in 10 mM PBS (pH 7.4) and then added to Caco-2 monolayers with an MOI at 10. For bacterial adhesion, cells were washed after 30 min post-infection, and adherent bacteria were isolated and enumerated by plating on BHI agar plates. For bacterial invasion, cells were washed with PBS after 30 min infection and incubated with DMEM, containing gentamicin at a final concentration of 50 μg/mL for 60 min to kill extracellular bacteria. Cells were then washed and lysed, and viable bacteria were enumerated as described above. For bacterial proliferation, the remaining cells were subjected to further incubation with DMEM, containing 5 μg/mL gentamicin and 10% FBS for 6 or 12 h. Viable bacteria were enumerated as described above.

### 2.12. Plaque Assay on L929 Fibroblast Cells

The plaque assay was carried out on mouse L929 fibroblast cells, as previously described [29]. Briefly, cell monolayers were maintained in high-glucose (4.5 g/L D-glucose) DMEM medium plus 15% (*v*/*v*) FBS. Cells plated at a density of 1 × 10^6^ cells per well in a six-well dish were infected with *L. monocytogenes* at an MOI at about 0.2 at 37 °C with 5% CO_2_ for 60 min. Gentamicin was added at a final concentration of 100 μg/mL to kill the extracellular bacteria. The infected cells were washed twice with 10 mM PBS (pH 7.4) and then overlaid with an equal volume of DMEM medium, containing 0.7% low-melting point agarose and 10 μg/mL gentamicin. Following a further 72-h incubation at 37 °C, cells were finally fixed with 4% paraformaldehyde for 20 min and stained with crystal violet. The numbers of plaques were measured by Adobe Photoshop software for each sample. The plaque number of the wild-type strain was set as 100%.

### 2.13. Virulence in Mouse Model

ICR mice (female, 18–22 g) were purchased from Zhejiang Academy of Medical Sciences and housed in the Laboratory Animal Center of Zhejiang A&F University. Animal experiments were performed in accordance with the Regulations for the Administration of Affairs Concerning Experimental Animals, approved by the Institutional Animal Care and Use Committee of Science Technology Department of Zhejiang Province (Permit Number: SYXK-2018-0010). The mice were maintained under the standard conditions of a 12 h light-dark cycle at 20–22 °C and relative humidity of 50–60% in the animal resource facility for at least 1 week prior to their use in experiments. The mice were anesthetized with an intraperitoneal injection of sodium pentobarbital (30 mg/kg body weight) prior to being subjected to experimental procedures. Specifically, the mice were infected intraperitoneally with 2 × 10^6^ CFU bacteria and were euthanized at 24 and 48 h post-infection, and the spleens and livers were harvested and homogenized. The numbers of viable bacteria in each organ were determined by plating onto BHI agar plates. For animal survival experiments, mice were infected intraperitoneally with 2 × 10^6^ CFU bacteria and monitored for up to 7 days post infection. Animal survival curves were plotted by using the Kaplan–Meier method, and differences in survival were determined by using the Log-Rank test. 

### 2.14. Statistical Analysis

All experiments were repeated three times. Data were analyzed using the two-tailed homoscedastic Student’s *t*-test. Differences with *p*-values < 0.05 were considered as statistically significant.

## 3. Results

### 3.1. LmAmpII is an Active M29 Aminopeptidase with a Wide Substrate Specificity

**Figure 1:**
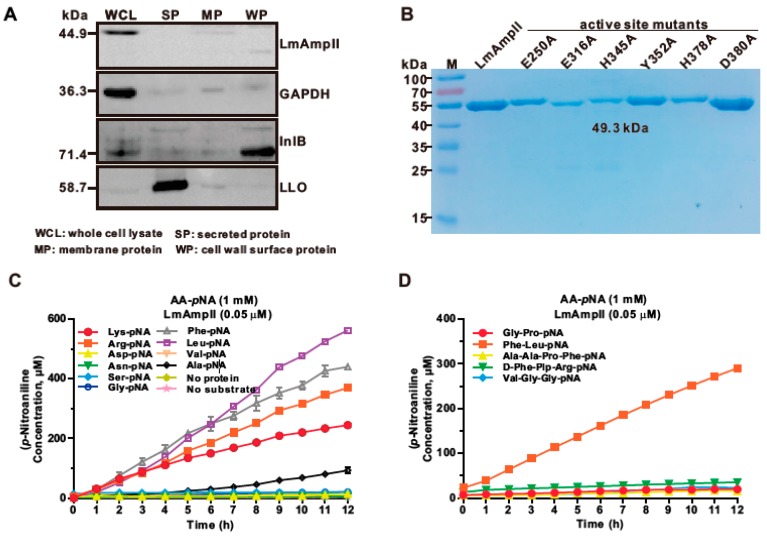

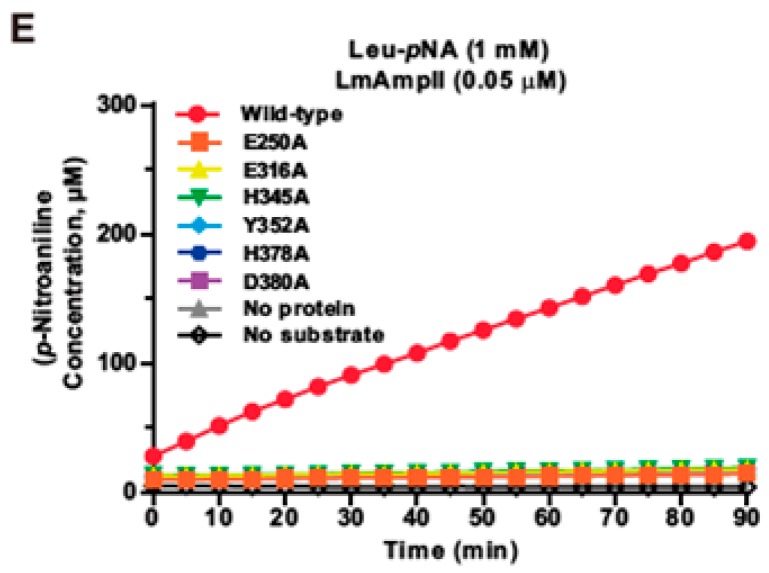
The cytoplasmic putative aminopeptidase II (LmAmpII) is an active aminopeptidase II of the M29 family with a wide range of substrate specificities. (**A**) Localization of LmAmpII *in L. monocytogenes*. Proteins were separated through a 12% SDS-PAGE and immunoblotted with α- LmAmpII, α-InlB, α-LLO, or α-GAPDH antisera. The predicted molecular weight of each protein is indicated on the left. (**B**) SDS-PAGE analysis of the recombinant LmAmpII and its mutant variants with single site-directed mutagenesis at the predicted active sites. (**C**,**D**) Enzymatic activity of LmAmpII using Lys-, Arg-, Asp-, Asn-, Ser-, Gly-, Leu-, Phe-, Val-, or Ala-*p*NA and polypeptides Gly-Pro-, Ala-Ala-Pro-Phe-, D-Phe-Pip-Arg-, or Val-Gly-Gly-*p*NA as substrate at a concentration of 1 mM in 25 mM Tris-HCl (pH 7.4) buffer. (**E**) Aminopeptidase activity of the wild-type LmAmpII and its mutant variants (E250A, E316A, H345A, Y352A, H378A, and D380A) using Leu-*p*NA as a substrate in 25 mM Tris-HCl (pH 7.4) buffer. Data shown represent mean ± SD.

LmAmpII has been predicted as a member of the M29 family [12], but the biological roles of which have not been well characterized to date. We found that LmAmpII is predominantly localized within bacterial cytosol (Figure 1A). The recombinant LmAmpII and its active site mutants were overexpressed in *E. coli* and then purified to homogeneity by nickel chelated affinity column chromatography (Figure 1B). Enzymatic activity of aminopeptidase was determined by measuring spectrophotometric detection of *p*NA, released by hydrolysis of Arg-, Leu-, Lys-, Asp-, Asn-, Ser-, Gly-, Phe-, Val-, or Ala-*p*NA as the substrate. As indicated, the recombinant aminopeptidase exhibited a rather broad substrate range and was capable to catalyze the release of *p*NA from Leu-, Phe-, Arg-, Lys-, or Ala-*p*NA with different efficiencies, while no activities were observed against Asp-, Ser-, Gly-, Val-, and Asn-*p*NA (Figure 1C). Significantly, LmAmpII showed the highest activity towards Leu-*p*NA, followed by Phe-*p*NA, Arg-*p*NA, and Lys-*p*NA, with the lowest activity towards Lys-*p*NA (Figure 1C). Interestingly, as for the polypeptide substrates, LmAmpII had a strong ability to hydrolyze Phe-Leu-*p*NA, but no activity was observed when it was incubated with remaining substrates, Gly-Pro-*p*NA, Ala-Ala-Pro-Phe-*p*NA, D-Phe-Pip-Arg-*p*NA, and Val-Gly-Gly-*p*NA (Figure 1D). These data implied that LmAmpII was able to degrade the polypeptides only composed of the amino acids that were considered as the appropriate substrates for this enzyme. The present findings indicate that *L. monocytogenes* LmAmpII is a functional M29 aminopeptidase with a broad substrate specificity with higher preference for leucine and arginine.

### 3.2. Site-Directed Mutagenesis Reveals That Key Residues in LmAmpII are Involved in Aminopeptidase Activity

The bioinformatic analysis suggested that E250, E316, H345, Y352, H378, and D380 could be the corresponding catalytic residues for LmAmpII. To establish that the predicted active sites are responsible for the measured aminopeptidase activity, site-directed mutagenesis targeted at these residues was undertaken to mutate them to alanines. These mutants with single amino acid substitution (E250A, E316A, H345A, Y352A, H378A, and D380A) were overproduced in *E. coli*, and their aminopeptidase activities were then tested. Single mutation at any of these residues completely forfeited its enzymatic activity using Leu-pNA as the substrate (Figure 1E), confirming that these residues play an essential role in aminopeptidase activity of LmAmpII.

### 3.3. LmAmpII Is Active in a Wide Range of pHs and Temperatures and can be Strongly Activated by Metal Ions

**Figure 2:**
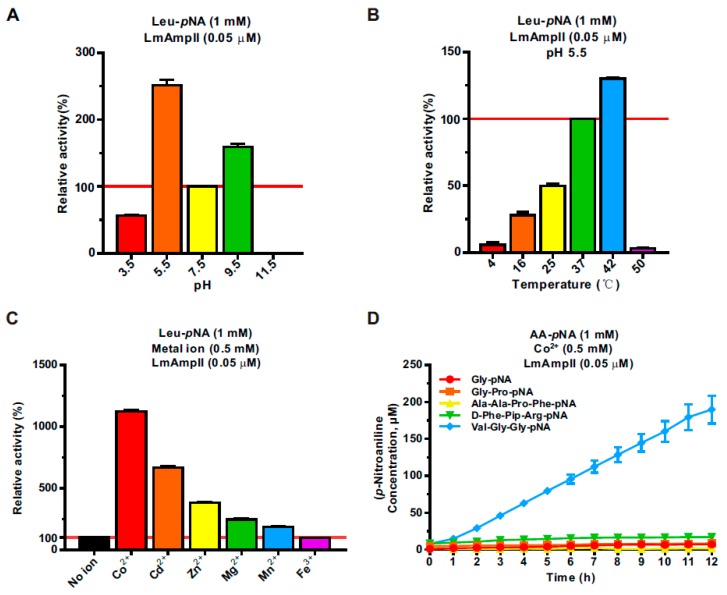
Biochemical characterization of LmAmpII. (**A**) Effect of pH on aminopeptidase activity. The enzymatic assays were performed over a pH range from 3.5 to 11.5 in 25 mM Tris-HCl buffer using Leu-*p*-nitroaniline (Leu-*p*NA) as a substrate. (**B**) Effect of temperature on aminopeptidase activity. The enzymatic assays were performed in 25 mM Tris-HCl (pH 7.4) buffer over a temperature range from 4–50 °C using Leu-*p*NA as a substrate. (**C**) Effect of metal ions on aminopeptidase activity. Activity was assayed in the presence of different metal ions (Co^2+^, Cd^2+^, Zn^2+^, Mg^2+^, Mn^2+^, or Fe^3+^, each at 0.5 mM) using Leu-*p*NA as a substrate. (**D**) Effect of Co^2+^ on aminopeptidase activity against the inert polypeptide substrates. Activity was assayed in the presence of using the polypeptides Gly-Pro-, Ala-Ala-Pro-Phe-, D-Phe-Pip-Arg-, or Val-Gly-Gly-*p*NA as a substrate at a concentration of 1 mM in 25 mM Tris-HCl (pH 7.4) buffer. Data shown represent mean ± SD. The red lines in (**A**), (**B**), and (**C**) indicate 100% relative enzymatic activity.

Considering the thermal stability and acid resistance of some aminopeptidases, we determined the optimum reaction conditions for hydrolysis activity of LmAmpII. Effects of pH and temperature on LmAmpII peptidase activity were examined spectrophotometrically using Leu-*p*NA as a substrate. The purified recombinant enzyme exhibited non-inactivated activity in a wide range of pH conditions from 3.5 to 9.5, with optimum activity at pH 5.5 (Figure 2A), suggesting that the activity of LmAmpII was not sensitive to pH. At the optimum pH at 5.5, we further determined thermal sensitivity of this enzyme. Interestingly, LmAmpII exhibited the highest activity at 42 °C and the enzymatic activity was almost completely lost when the temperature was higher than 50 °C or lower than 4 °C (Figure 2B). The pH and temperature adaptability of LmAmpII was almost consistent, with the existing fact that *L. monocytogenes* is well-adapted to a wide range of environmental stress conditions, including heat and acidity stress [30], suggesting an interesting characteristic of this peptidase. As LmAmpII belongs to the metallopeptidase that has wide-ranging preference for metal ions, we therefore determined the metal ion dependency of LmAmpII. As indicated, LmAmpII displayed enhanced catalytic activity in the presence of metal ions, including Co^2+^, Cd^2+^, Zn^2+^, Mg^2+^, and Mn^2+^, while incubation of Fe^3+^ did not affect activation of this enzyme. Significantly, the maximum activity was observed in the presence of Co^2+^ (11.2-fold), followed by Cd^2+^, Zn^2+^, Mg^2+^, and Mn^2+^ (Figure 2C). To our surprise, the polypeptide substrate Val-Gly-Gly-*p*NA, considered as an inappropriate substrate for LmAmpII, could be efficiently degraded by this peptidase in the presence of Co^2+^ (Figure 2D). Collectively, these results strongly indicated that LmAmpII is a cobalt-dependent metalloenzyme and is active in a broad range of pHs and temperatures. 

### 3.4. Kinetic Properties of LmAmpII in the Presence of Various Substrates

**Figure 3:**
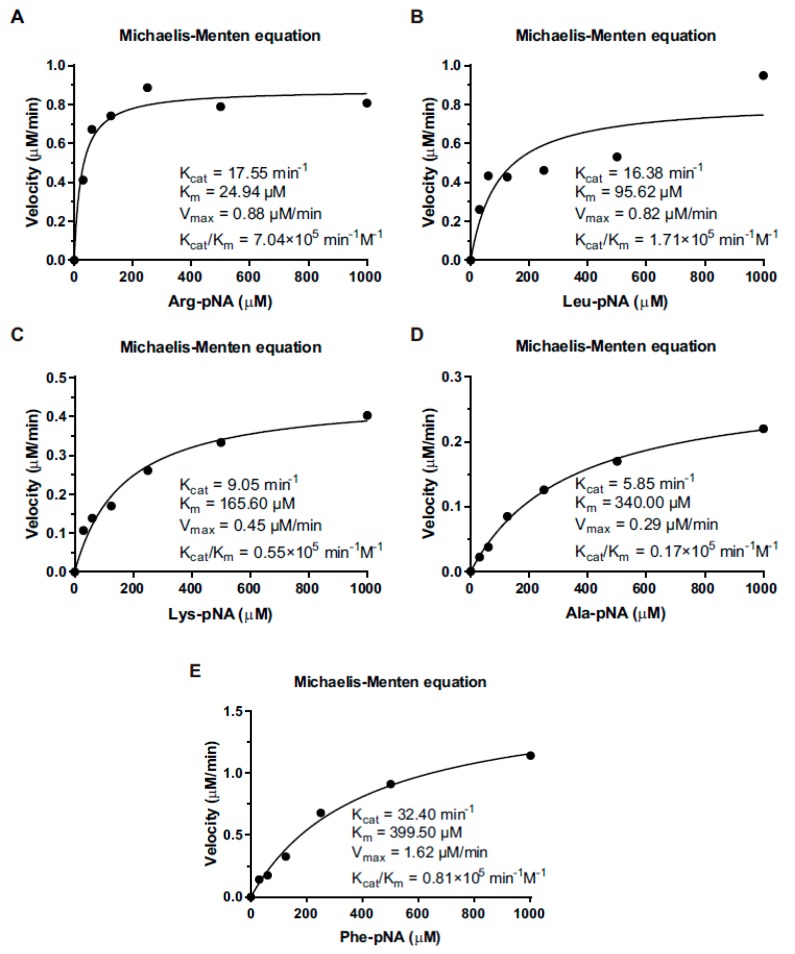
The Michaelis–Menten kinetic parameters of LmAmpII. The Michaelis–Menten curves of LmAmpII were plotted, and the *K*_m_, *V*_max_, *K*_cat_, and *K*_cat_/*K*_m_ values were determined for each substrate. (**A**) Arg-*p*NA, (**B**) Leu-*p*NA, (**C**) Lys-*p*NA, (**D**) Ala-*p*NA, or (**E**) Phe-*p*NA. These assays were carried out using the specific substrate at a concentration range of 0–1000 μM in 25 mM Tris-HCl (pH 7.4), containing 0.05 μM enzyme. Data of each black dot shown represent mean ± SD.

To assess the enzyme-substrate preference, the recombinant aminopeptidase was incubated with the above selected substrates. Plotting of enzymatic velocity against substrate concentrations yielded a curve that fit the classical Michaelis–Menten model: *K*_m_, *V*_max_, *K*_cat_, and *K*_cat_/*K*_m_ values of the enzyme for each substrate. Calculated *K*_m_ values were 24.94, 95.62, 165.6, 340, and 399.6 μM for Arg-, Leu-, Lys-, Ala, and Phe-*p*NA, respectively. The corresponding enzyme efficiency ratio *K*_cat_/*K*_m_ values were 7.04 × 10^5^ min^−1^ M^−1^ (Figure 3A), 1.71 × 10^5^ min^−1^ M^−1^ (Figure 3B), 0.55 × 10^5^ min^−1^ M^−1^ (Figure 3C), 0.17 × 10^5^ min^−1^ M^−1^ (Figure 3D), and 0.81 × 10^5^ min^−1^ M^−1^ (Figure 3E). In general, the enzyme showed relatively higher activity on substrates with hydrophobic amino acids than hydrophilic amino acids, but there are exceptions for arginine and lysine, which were considered as preferential substrates.

### 3.5. LmAmpII is Required for In Vitro Growth in Synthetic Media, but not in Rich Media

**Figure 4:**
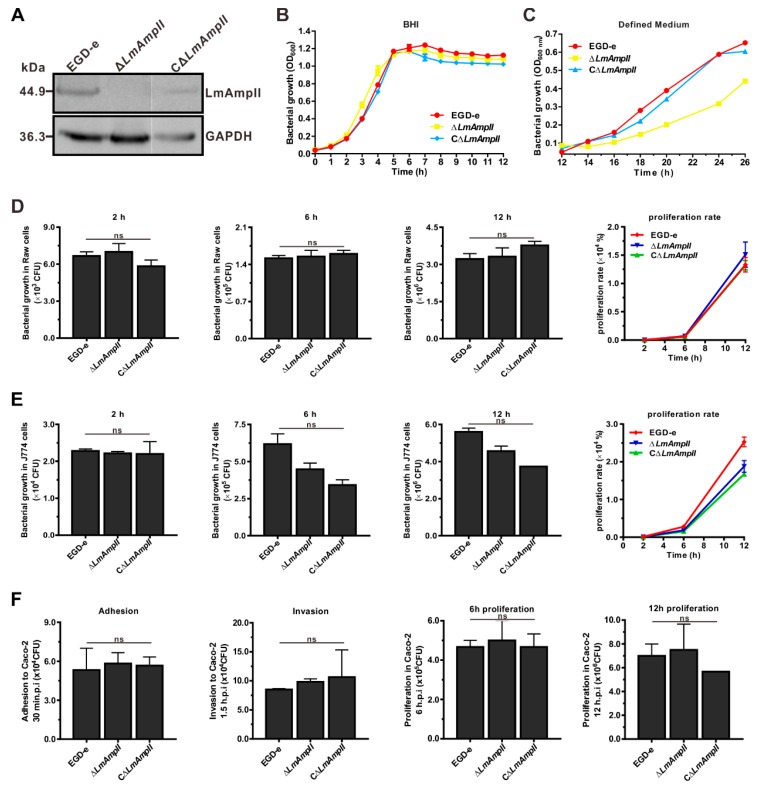
LmAmpII is required for bacterial growth in a chemically defined medium for optimal growth of *L. monocytogenes*, but not for intracellular infection and virulence. (**A**) Construction of the LmAmpII deletion and complemented mutants, and protein expression was detected by Western blotting. (**B**,**C**) Growth curves of *L. monocytogenes* bacteria in the brain heart infusion (BHI) medium (**B**) and chemically defined medium (**C**). (**D**,**E**) Intracellular growth of *L. monocytogenes* in murine-derived Raw264.7 (**D**) and J774A.1 (**E**) macrophages. Gentamicin (50 μg/mL) was added 30 min post-infection. Cells infected with *L. monocytogenes* strains that were lysed at the indicated time points (2, 6, and 12 h), and viable bacteria were numbered by serial plating on BHI agar plates. (**F**) Adhesion, invasion, and proliferation of *L. monocytogenes* in human intestinal epithelial cells Caco-2. Cells infected with bacteria were lysed at the indicated time points, and viable bacteria were serially plated on BHI agar plates. The number of recovered bacteria shown that were able to invade cells and survive represent the mean ± SD for each strain. ns, not significant.

Previously, it was proposed that aminopeptidase was involved in bacterial growth in rich media [5,31]. To illustrate whether LmAmpII was involved in this cellular process, growth of wild-type and the generated LmAmpII mutant strains (Figure 4A) was assessed at 37 °C on rich media BHI and in the *L. monocytogenes* chemically defined media. For these mutant strains, no significant differences were observed for bacterial growth in BHI, while growth in chemically defined media was obviously affected by the absence of LmAmpII (Figure 4B,C). These results indicate that the aminopeptidase LmAmpII is involved in growth on the nutrient-poor medium, but are not essential for growth in rich conditions. Considering the above-determined fact that LmAmpII is efficiently able to catalyze hydrolysis of a wide range of substrates which are fundamentally essential amino acids for bacterial growth metabolism, and we suggest that LmAmpII contributes to *L. monocytogenes* growth on synthetic media, perhaps because of the limited supply of some amino acids on a less rich medium. 

### 3.6. LmAmpII Is Not Necessary for Bacterial Infection and Virulence

**Figure 5:**
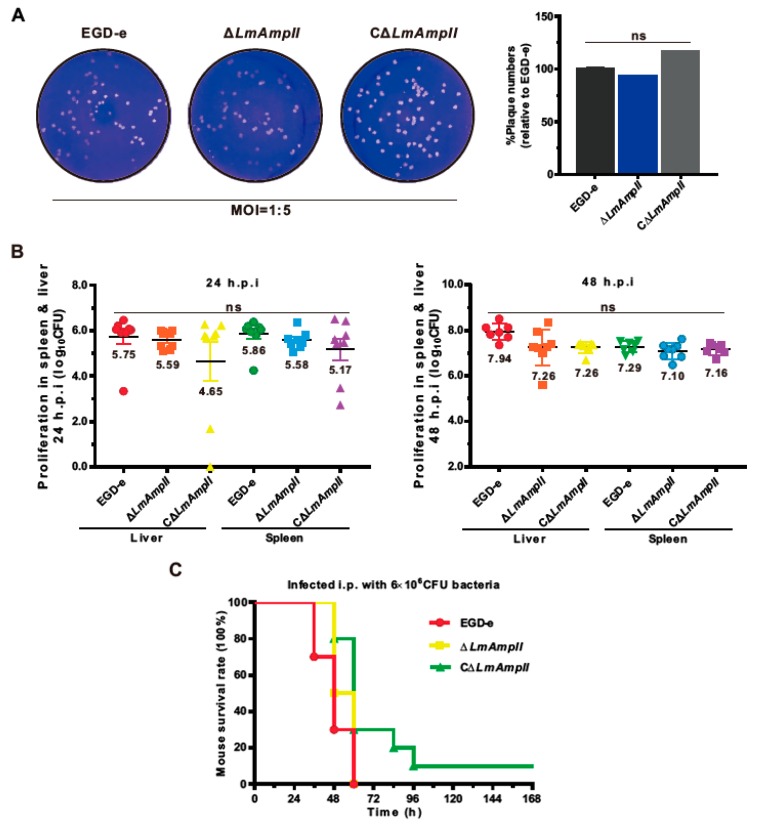
LmAmpII was not essential for *L. monocytogenes* virulence in the mouse model. (**A**) Plaque assay performed on L929 fibroblast monolayers infected by *L. monocytogenes*. The plaque numbers of the mutant strains were indicated as a percentage of those formed by the wild-type strain. (**B**) Proliferation of *L. monocytogenes* in mice organs. Mice were inoculated intraperitoneally with 2 × 10^6^ CFU bacteria, and animals were euthanized 24 and 48 h post-infection (h.p.i), and numbers of bacteria colonized in organs shown represent the mean ± SD of the log_10_cfu per organ for each group. (**C**) Kaplan–Meier survival of the infected ICR mice over time. The mice were challenged intraperitoneally with 2 × 10^6^ CFU bacteria and monitored for up to 7 days post-inoculation. Data shown represented as the percentage survival over time and significance was determined via a Log-rank test. ns, not significant.

As we previously determined a critical role of an M29 family peptidase AmpT in *Listeria* infection and pathogenicity, similar functions of LmAmpII were further evaluated in the cellular and murine infection model. For the wild-type and mutant strains, results showed no significant difference in their ability to multiply and survive, both inside RAW264.7 and J774 macrophages (Figure 4D,E). The ability to adhere to or colonize epithelial cells is an essential and prerequisite trait for *Listeria*. The invasion and adhesion abilities of wild-type and LmAmpII-deficient strains were analyzed on human intestinal epithelial Caco-2 cells. There was no marked difference among these strains in invading Caco-2 cells (Figure 4F). The plaque assay performed on the murine fibroblast L929 further showed that these strains were able to produce a similar number of plaques, suggesting that the *LmAmpII* gene was not essential for cell-to-cell spreading ability (Figure 5A). Moreover, the equivalent bacterial burdens at 24 or 48 h post-infection in both livers and spleens were observed in mice inoculated with *L. monocytogenes* bacteria (Figure 5B), and the mouse survival test showed that mortality of infected mice did not differ significantly 7 days post-infection (Figure 5C). These data clearly indicate that LmAmpII does not participate in bacterial intracellular infection, and more importantly, does not contribute to virulence on the mice model of *Listeria*.

## 4. Discussion

Aminopeptidases are known to contribute to bacterial physiology and have been well characterized in several pathogenic bacterial species [32]. The gram-positive bacterial aminopeptidases have previously been demonstrated for their roles in amino acid metabolism and catabolizing peptides to serve as a nitrogen source. Recently, the importance of these enzymes has been emphasized, as it has emerged that aminopeptidases play a critical role in bacterial pathogenesis [33]. In our previous study, for the first time, we identified a functional aminopeptidase T (AmpT, encoded by *lmo1603*) of the M29 family that has been demonstrated to act as a novel intracellular virulence factor essential in the successful establishment of *L. monocytogenes* infection, but not required for bacterial in vitro growth [8]. Here in the present study, we again clarified that *L. monocytogenes* additionally possess another M29 family aminopeptidase, LmAmpII, which exhibits the classical properties of this family and was required for the nutrition metabolism of *L. monocytogenes*, but not required for bacterial infection and pathogenicity. Although these two aminopeptidases of *Listeria* were predicted as belonging to the same family, the previous and present data suggest that the two peptidases play different roles in bacterial physiology during *Listeria* environmental adaption and host infection. *L. monocytogenes* AmpII might participate in the bacterial in vitro environmental adaption, while the AmpT mainly contributes to in vivo infection.

All the M29 aminopeptidases contain a highly conserved catalytic triad (EEHYHD), suggesting a common reaction mechanism [16,34]. The crystal structures and catalytic mechanism of the three members (AmpT, PepS, and AmpS) of M29 aminopeptidases have already been elucidated [13,31,34]. For example, the active sites from *Staphylococcus aureus* AmpS are located at opposite ends of a large internal cavity. Two metal ions with full occupancy and a third metal ion with low occupancy are present in the active site. A water molecule and E319 serve as bridging ligands to the two metals with full occupancy. Additionally, E253 and H348 coordinate one of these metal ions, and H381 and D383 coordinate the other one. Involvement of the metals in weak metal-donor interactions to a water molecule and to Y355 are found [13]. Odintsov and colleagues further demonstrated that the AmpT from *Thermus thermophilus*, like AmpS, forms highly elongated dimers in the solution, and AmpT can take many different conformations, from fully closed to nearly open, with an almost exposed active site. This domain movement is likely to be a key feature of AmpT and its homologues [34]. Based on these known structures, we predicted the 3D structures of the two M29 aminopeptidases of *L. monocytogenes*, showing that they share a very high structure similarity. Bacterial AmpIIs are most commonly found in the cytosol and generally act as homo-dimeric enzymes. In agreement with the literature, we found that LmAmpII is also localized to the bacterial cytosol and is predicted as an oligomer metallopeptidase formed by two monomers. Our data suggests that the active sites of LmAmpII incorporate the conserved catalytic sites of the M29 family, and the residues E250, E316, H345, Y352, H378, and D380 are the corresponding active sites, as confirmed by site-directed mutagenesis, revealing that single mutation at any one of these residues completely abolished the enzymatic activity. Members of the M29 family exhibit a rather broad substrate specificity, with a preference for hydrophobic residues, such as Leu, Val, Phe, or Tyr at the amino terminus [13]. We determined here that LmAmpII was able to efficiently catalyze the hydrolysis of the residue Leu, Phe, Arg, or Lys from the N-terminus of peptide, especially with the higher substrate preference for Arg and Leu. Moreover, peptidases in the family M29 have cocatalytic metal ions, and the member AmpII has been shown to possess cocatalytic cobalt ion [35,36,37]. In agreement with our previous findings on AmpT [8], LmAmpII activity required the presence of a wide range of metals and exhibits the maximum activity in the presence of Co^2+^. A previous study clearly determined that the electronic nature of cobalt and manganese ions activates water molecules, thus influencing the generation of the intermediate state, which consequently accelerates the reaction rate [38].

In this study, our data demonstrate an absence of AmpII, which markedly affected bacterial growth in the chemically defined medium, a less-rich media for *Listeria spp.* growth, and no obvious influence was found on the growth in rich media, such as a brain heart infusion (BHI). On the contrary, we previously reported that another M29 aminopeptidase AmpT of *L. monocytogenes* was dispensable for growth in the *Listeria* chemically-defined medium [8]. Together, these results indicate that an absence of AmpII is not totally compensated by the other peptidases present in *L. monocytogenes*, which could supply the demand for intracellular amino acids required for bacterial development, such as protein synthesis. Various effects of bacterial aminopeptidases on growth have been well elucidated to date. The *Mesorhizobium* AmpT has been found not required for growth in synthetic media but for biofilm production [6], whereas, in *S. thermophilus*, hawse have come to a conclusion that the M29 aminopeptidase PepS is involved in bacterial growth, both on rich media and chemically-defined media [5]. On the other hand, in *L. lactis*, inactivation of peptidases PepN, PepX, PepC, PepT, or PepO, or of all five, has no significant effect on bacterial growth on a rich medium [39]. Therefore, it is not surprising that Ampll and AmpT peptidases, although within the same family, differently contributed to in vitro growth of *L. monocytogenes*. More importantly, we further demonstrated that LmAmpII was dispensable for *Listeria* infection and virulence, contrarily to AmpT. 

As previously described, members from the M17 family leucine aminopeptidase (LAP) have been investigated in bacteria with virulence association. Andre and co-workers suggested that the LAP of *Mycobacterium tuberculosis* participates in important metabolic pathways of this pathogen necessary for its survival and virulence and consequently may be a promising target for new anti-TB drugs [40]. The LAP of *S. aureus* is an intracellular enzyme and is not required for bacterial growth but is required for virulence of this pathogen [31,33]. In addition, Luckett and colleagues have demonstrated that the M28 family arginine-specific aminopeptidase acts as an important virulence factor in *Pseudomonas aeruginosa* in vivo virulence [9]. However, so far, most of the research on the M29 family aminopeptidases has been restricted to their biochemical properties and to biophysical characterization. The M29 family encompasses AmpT, AmpII, PepS, and AmpS [12]. In *Streptococcus thermophilus*, PepS aminopeptidase is involved in peptidoglycan metabolism. In a previous study, we identified a functional aminopeptidase T of the M29 family that has been demonstrated to act as a novel intracellular virulence factor essential in the successful establishment of *L. monocytogenes* infection, but is not required for bacterial in vitro growth [8]. As a multifaceted bacterial pathogen, *L. monocytogenes* is well-adapted to both life in the outside environment and life inside the cytosol of eukaryotic host cells and is capable of easily making a transition from a saprophyte to an intracellular pathogen [41]. Recent studies suggest that *L. monocytogenes* has many sophisticated mechanisms to mediate this saprophyte-to-cytosolic pathogen transition, through the precise role-division of many virulence factors [42]. Our results suggest that these two aminopeptidases have complementary functions that allow *L. monocytogenes* to rapidly adapt to different environmental conditions, and that AmpII is only required for survival in poor nutritional conditions in the environment. Together with the findings in our previous and present study, these results reveal that *L. monocytogenes* produces dimeric cytosol aminopeptidases that are members of the M29 Mpl family with classical enzymatic properties. LmAmpII is able to efficiently hydrolyze a wide range of amino acids, preferably Arginine and Leucine. More importantly, this aminopeptidase was required for bacterial in vitro growth but not for intracellular infection, as well as bacterial pathogenicity. These data demonstrate that *L. monocytogenes* is engineered with two aminopeptidases that play an important role in its particular ability to adapt to extracellular and intracellular environmental constraints and that the nutritional and metabolic roles of these aminopeptidases is not always associated with virulence. The precise mechanisms by which the aminopeptidases interfere with the cellular functions need to be further elucidated in the future research.

## 5. Conclusions

We conclude that LmAmpII, an M29 family aminopeptidase, can efficiently hydrolyze a wide range of substrates and is required for in vitro bacterial growth, which lays a foundation for in-depth investigations of aminopeptidases as potential targets to defend *Listeria* infection.

## Figures and Tables

**Table 1:** PCR Primers used in this study. Nucleotides introduced to create restriction enzyme sites are underlined.

Name	Sequence (5’-3’)	Description
LmAmpII-exp-F	CGGGGTACCATGACAGTATTTAGTGAAAAGTTAGAAAAGTATGC	Recombinant LmAmpII expression
LmAmpII-exp-R	CGCGGATCCTTAGAACGCCCAGTCGCCTTTA	
LmAmpII-a-front	AGTTTTTCTGAAGACATTTATAATAGAAGGTATCAG	Construction of LmAmpII null mutant
LmAmpII-a	CGCGGATCCTAATACACAAGAAATTGCCGACATTTTAG	
LmAmpII-b	TTAAAAAAAGGAAATTAATCACTCCAATCTTTTTATTCAATACG	
LmAmpII-c	GAGTGATTAATTTCCTTTTTTTAAACTTATGCCTTTTGC	
LmAmpII-d	CCCAAGCTTATCATATAGAACACCGAATAAATATGTGTCC	
LmAmpII-g	GACGAGCTCCGAAAGCTACGCGAAAACTTTCGTTG	Complementation of the LmAmpII deletion
LmAmpII-h	CGCGGATCCTTAGAACGCCCAGTCGCCTTTACG	
E250A-fwd	TCCGCACAACAGAAAACTGCTTCTGTTGGCATATTGG	Recombinant LmAmpII E250A expression
E250A-rev	CCAATATGCCAACAGAAGCAGTTTTCTGTTGTGCGGA	
E316A-fwd	CTGGAACTAGCGCCACTGCACCTAAATAGTGTGAG	Recombinant LmAmpII E316A expression
E316A-rev	CTCACACTATTTAGGTGCAGTGGCGCTAGTTCCAG	
H345A-fwd	CATACGCACTACCAATTGCTAAGGCGTTAGAAGCATTTTCGTCAAATA	Recombinant LmAmpII H345A expression
H345A-rev	TATTTGACGAAAATGCTTCTAACGCCTTAGCAATTGGTAGTGCGTATG	
Y352A-fwd	GCCACCTTTAACATTAAATGCAGCCGCACTACCAATTGCTAAGTGG	Recombinant LmAmpII Y352A expression
Y352A-rev	CCACTTAGCAATTGGTAGTGCGGCTGCATTTAATGTTAAAGGTGGC	
H378A-fwd	AATCATGAAATCAACGGCTGTTAAACTATTGTTCACACCAGCTGCTTCT	Recombinant LmAmpII H378A expression
H378A-rev	AGAAGCAGCTGGTGTGAACAATAGTTTAACAGCCGTTGATTTCATGATT	
D380A-fwd	TTTCAGAAGAACCAATCATGAAAGCAACGTGTGTTAAACTATTGTTC	Recombinant LmAmpII D380A expression
D380A-rev	GAACAATAGTTTAACACACGTTGCTTTCATGATTGGTTCTTCTGAAA	

## Abbreviations

AmpII	Aminopeptidase type II
Mpl	Metalloprotease
AmpS	Aminopeptidase S
PepP	X-prolyl aminopeptidase
BHI	Brain heart infusion
LB	Luria–Bertani broth
SP	Secreted proteins
TCA	Trichloroacetic acid
WCL	Whole cell lysates
MP	Membrane proteins
WP	Cell wall proteins
IPTG	Isopropyl β-D-1-thiogalactopyranoside
AAs-*p*NA	Amino acids-p-nitroaniline
DMEM	Dulbecco’s modified eagle medium
FBS	Fetal bovine serum
AmpT	Aminopeptidase T
LAP	Leucine aminopeptidase
